# Supplementary material for: Comprehensive Evaluation of a 1021-Gene Panel in FFPE and Liquid Biopsy for Analytical and Clinical Use
Source: Int J Mol Sci. 2025 Jun 20;26(13):5930. doi: 10.3390/ijms26135930 (PMC12249698; doi:10.3390/ijms26135930)
Supplement: Supplementary file 1 [file ijms-26-05930-s001.zip › SUPPLEMENTARY TABLE 3.pdf]

## 12.e. Genes Analyzed

| 312 genes including all exon regions and available for detecting SNV / Indel / CNV | | | | | | | | | |
|---|---|---|---|---|---|---|---|---|---|
| ABL1 | ACVR1B | AKT1 | AKT2 | AKT3 | ALK | APC | AR | ARAF | ARID1A |
| ARID1B | ARID2 | ASXL1 | ATM | ATR | ATRX | AURKA | AURKB | AXIN1 | AXIN2 |
| AXL | B2M | BAP1 | BARD1 | BCL2 | BCL2L1 | BCOR | BLM | BMPR1A | BRAF |
| BRCA1 | BRCA2 | BRD4 | BRIP1 | BTK | CARD11 | CASP8 | CBFB | CBL | CCND1 |
| CCND2 | CCND3 | CCNE1 | CD274 | CDC73 | CDH1 | CDK12 | CDK4 | CDK6 | CDK8 |
| CDKN1A | CDKN1B | CDKN2A | CDKN2B | CDKN2C | CEBPA | CHEK1 | CHEK2 | CIC | CREBBP |
| CRKL | CSF1R | CTCF | CTNNA1 | CTNNB1 | CUL3 | CYLD | DAXX | DDR1 | DDR2 |
| DICER1 | DNMT3A | DOT1L | EGFR | EIF1AX | EMSY | EP300 | EPAS1 | EPCAM | EPHA2 |
| EPHA3 | EPHA5 | EPHB1 | EPHB6 | ERBB2 | ERBB3 | ERBB4 | ERCC1 | ERCC3 | ERCC4 |
| ERCC5 | ERG | ERRFI1 | ESR1 | EXT1 | EXT2 | EZH2 | FAM123B | FAM175A | FANCA |
| FANCC | FANCD2 | FANCE | FANCF | FANCG | FANCL | FANCM | FAS | FAT1 | FAT2 |
| FBXW7 | FGF19 | FGF3 | FGF4 | FGFR1 | FGFR2 | FGFR3 | FGFR4 | FH | FLCN |
| FLT1 | FLT3 | FLT4 | FOXA1 | FOXL2 | FOXP1 | FUBP1 | GALNT12 | GATA3 | GNA11 |
| GNAQ | GNAS | GRIN2A | GRM3 | HDAC1 | HGF | HNF1A | HOXB13 | HRAS | IDH1 |
| IDH2 | IFNG | IFNGR1 | IGF1R | IKBKE | IKZF1 | IL7R | INPP4B | IRF2 | IRS2 |
| JAK1 | JAK2 | JAK3 | JUN | KDM5A | KDM5C | KDM6A | KDR | KEAP1 | KIT |
| KRAS | LRP1B | MAF | MAP2K1 | MAP2K2 | MAP2K4 | MAP3K1 | MAPK1 | MAX | MCL1 |
| MDM2 | MDM4 | MED12 | MEF2B | MEN1 | MET | MITF | MLH1 | MLH3 | MLL |
| MLL2 | MLL3 | MPL | MRE11A | MS4A1 | MSH2 | MSH3 | MSH6 | MST1R | MTOR |
| MUTYH | MYC | MYCL1 | MYCN | MYD88 | NBN | NCOR1 | NF1 | NF2 | NFE2L2 |
| NFKB1A | NKX2-1 | NOTCH1 | NOTCH2 | NOTCH3 | NPM1 | NRAS | NSD1 | NTHL1 | NTRK1 |
| NTRK2 | NTRK3 | PALB2 | PARK2 | PARP1 | PAX5 | PBRM1 | PCK1 | PDCD1 | PDCD1LG2 |
| PDGFRA | PDGFRB | PDK1 | PIK3CA | PIK3CB | PIK3CG | PIK3R1 | PIK3R2 | PMS1 | PMS2 |
| POLD1 | POLE | POT1 | PPP2R1A | PRDM1 | PRKAR1A | PTCH1 | PTCH2 | PTEN | PTPN11 |
| PTPRD | RAC1 | RAD50 | RAD51 | RAD51B | RAD51C | RAD51D | RAD52 | RAD54L | RAF1 |
| RARA | RB1 | RBM10 | RECQL | RECQL4 | RET | RHOA | RICTOR | RINT1 | RNF43 |
| ROS1 | RPTOR | RUNX1 | SDHA | SDHAF2 | SDHB | SDHC | SDHD | SERPINB3 | SERPINB4 |
| SETD2 | SF3B1 | SLX4 | SMAD2 | SMAD3 | SMAD4 | SMARCA4 | SMARCB1 | SMO | SOCS1 |
| SOX2 | SOX9 | SPOP | SRC | STAG2 | STAT3 | STK11 | SUFU | SYK | TBX3 |
| TCF7L2 | TERC | TET2 | TGFBR2 | TMEM127 | TMPRSS2 | TNFAIP3 | TNFRSF14 | TOP1 | TOP2A |
| TP53 | TSC1 | TSC2 | TSHR | U2AF1 | VEGFA | VHL | WRN | WT1 | XPO1 |
| XRCC2 | ZMAT3 | | | | | | | | |
| | | | | | | | | | |

| 38 genes including specific intron, promoter and fusion breakpoint regions and available for detecting gene rearrangement or fusion | | | | | | | | | |
|---|---|---|---|---|---|---|---|---|---|
| ALK | BCL2L11 | BRAF | BRCA1 | BRD4 | CD74 | EGFR | EML4 | ERG | ETV6 |
| EZR | FGFR1 | FGFR2 | FGFR3 | KIF5B | KIT | MAML2 | MET | MSH2 | MYC |
| MYCL1 | NCOA4 | NOTCH2 | NTRK1 | NTRK2 | NTRK3 | PDGFRA | RAF1 | RET | ROS1 |
| RSPO2 | SDC4 | SLC34A2 | TERT | TFE3 | TMPRSS2 | TPM3 | PMS2 | | |
| | | | | | | | | | |

| 709 genes including partial exon regions and available for detecting SNV / Indel | | | | | | | | | |
|---|---|---|---|---|---|---|---|---|---|
| ABCA13 | ABCB1 | ABCC1 | ABCC11 | ABCC2 | ABCG2 | ABL2 | ACACA | ACIN1 | ACTB |
| ACTG1 | ACTG2 | ACVR2A | ACVRL1 | ADAM29 | ADAMTS5 | ADCY1 | AFF1 | AFF2 | AFF3 |
| AHNAK | AKAP9 | ALB | AMOT | ANGPT1 | ANK3 | ANKRD11 | ANKRD30A | ANKRD30B | APEX1 |
| APOBEC3B | ARAP3 | ARFGEF1 | ARFGEF2 | ARHGAP29 | ARHGAP35 | ARID4B | ARID5B | ARNT | ASCL4 |
| ASH1L | ASMTL | ASPM | ASTN1 | ASXL2 | ATIC | ATP11B | ATP12A | ATP1A1 | ATP2B3 |
| BAZ2B | BBC3 | BBS9 | BCAS1 | BCL10 | BCL11A | BCL11B | BCL2A1 | BCL2L11 | BCL3 |
| BCL6 | BCL9 | BCORL1 | BCR | BIRC3 | BMPR2 | BNC2 | BPTF | BRD2 | BRD3 |
| BRSK1 | BRWD1 | BTLA | BUB1 | C15orf23 | C15orf55 | C1QA | C1S | C3orf70 | C7orf53 |
| C8orf34 | CACNA1E | CADM2 | CALR | CAMTA1 | CASP1 | CASQ2 | CBLB | CBR1 | CBR3 |
| CCDC168 | CCNA1 | CCNB3 | CCT3 | CCT5 | CCT6B | CD22 | CD33 | CD5L | CD74 |
| CDA | CDH11 | CDH18 | CDH23 | CDK13 | CHD1 | CHD1L | CHD4 | CHD6 | CHD8 |
| CHD9 | CHFR | CHI3L1 | CHN1 | CIITA | CLDN18 | CLP1 | CLSPN | CLTC | CNOT3 |
| CNOT4 | CNTN1 | CNTN5 | CNTNAP1 | CNTNAP5 | COL1A1 | COL2A1 | COL5A1 | COL5A2 | COL5A3 |
| COPS2 | CPS1 | CRIPAK | CRLF2 | CRNKL1 | CRTC1 | CSF1 | CSF3R | CSMD1 | CSMD3 |
| CSNK1A1 | CSNK1G3 | CTLA4 | CTNNA2 | CTNND1 | CUX1 | CXCR4 | CYBA | CYP19A1 | CYP1A1 |
| CYP1B1 | CYP2A13 | CYP2C8 | CYP2D6 | CYP3A4 | CYP3A5 | DCC | DDX3X | DDX5 | DEK |
| DHX35 | DHX9 | DIAPH1 | DIS3L2 | DLC1 | DMD | DNAH6 | DNAJB1 | DNM2 | DNMT1 |
| DNMT3B | DOCK2 | DOCK7 | DPYD | DRGX | DTX1 | DUSP22 | DYSF | E2F3 | EBF1 |
| ECT2L | EED | EEF1A1 | EGFL7 | EGR3 | EIF2AK3 | EIF2C3 | EIF3A | EIF4A2 | EIF4G3 |
| ELAC2 | ELF1 | ELF3 | ELMO1 | ELN | EME2 | EMID2 | EML4 | EPC1 | EPHA1 |
| EPHA4 | EPHA7 | EPHB2 | EPHB4 | EPOR | EPPK1 | EPS15 | ERBB2IP | ERCC2 | ESR2 |
| ETS1 | ETV1 | ETV5 | ETV6 | EWSR1 | EZR | F8 | FAM131B | FAM135B | FAM157B |
| FAM46C | FAM5C | FAP | FASLG | FAT3 | FAT4 | FCGR1A | FCGR2A | FCGR2B | FCGR3A |
| FCRL4 | FGF10 | FGF12 | FGF14 | FGF23 | FGF6 | FLG | FLI1 | FLNC | FMN2 |
| FN1 | FNDC4 | FOXA2 | FOXO1 | FOXO3 | FOXQ1 | FRMPD4 | FUS | FXR1 | FYN |
| FZD1 | G3BP1 | G3BP2 | GAB2 | GABRA6 | GATA1 | GATA2 | GFRAL | GIGYF1 | GKN2 |
| GLB1L3 | GLI1 | GLI2 | GLI3 | GMPS | GNA13 | GNG2 | GPC3 | GPR124 | GPS2 |
| GPX1 | GRB7 | GSK3B | GSTM5 | GSTP1 | GUSB | H3F3A | H3F3B | H3F3C | HCLS1 |
| HCN1 | HDAC4 | HDAC9 | HECW1 | HEY1 | HIST1H1C | HIST1H1D | HIST1H1E | HIST1H2AC | HIST1H2AG |
| HIST1H2AL | HIST1H2AM | HIST1H2BC | HIST1H2BD | HIST1H2BJ | HIST1H2BK | HIST1H2BO | HIST1H3B | HIST1H3C | HIST1H3D |
| HIST1H3F | HIST1H3G | HIST1H3H | HIST1H3I | HIST1H4I | HIST3H3 | HLA-A | HLA-B | HLA-C | HLF |
| HMCN1 | HNF1B | HNRPDL | HOXA11 | HOXA13 | HOXA3 | HOXA9 | HOXC13 | HOXD11 | HOXD13 |
| HSD3B1 | HSP90AA1 | HSP90AB1 | HSPA8 | HSPD1 | HSPH1 | ICK | ICOSLG | ID3 | IFITM3 |
| IGF1 | IGF2 | IGF2R | IGLL5 | IKZF2 | IKZF3 | IL10 | IL1RAPL1 | IL21R | IL6 |
| IL6ST | IMPG1 | ING1 | INHBA | INPP4A | INPPL1 | INSR | IRF4 | IRF6 | IRS1 |
| ITGB3 | ITK | ITSN1 | JARID2 | KALRN | KAT6A | KAT6B | KCNJ5 | KCNQ2 | KDM2B |
| KEL | KIF5B | KLF4 | KLHL6 | KLK1 | KRTAP5-5 | L3MBTL1 | LAMA2 | LATS1 | LATS2 |
| LCP1 | LEF1 | LGALS8 | LIFR | LPHN2 | LPP | LRP2 | LRP4 | LRP5 | LRP6 |
| LRRC7 | LRRK2 | LYN | LZTS1 | MACF1 | MAD1L1 | MAGI2 | MAML2 | MAML3 | MAP3K13 |
| MAPK3 | MCC | MCM3 | MDC1 | MECOM | MEF2C | MGA | MIB1 | MIOS | MKL1 |
| MLL4 | MLLT3 | MMP11 | MMP2 | MN1 | MNDA | MNX1 | MSH4 | MSN | MSR1 |
| MTHFR | MTRR | MUC5B | MYH11 | MYH14 | MYH9 | MYO3A | MYOD1 | NAP1L1 | NAV3 |
| NCAM2 | NCF2 | NCF4 | NCK1 | NCOA3 | NCOA4 | NCOR2 | NCSTN | NDUFA13 | NFATC4 |

| | | | | | | | | | |
|---|---|---|---|---|---|---|---|---|---|
| NFE2L3 | NKX3-1 | NLRC3 | NOD1 | NOS3 | NOTCH4 | NQO1 | NR1I2 | NR2F2 | NR4A2 |
| NRG1 | NRP2 | NRXN1 | NTM | NUMA1 | NUP107 | NUP210 | NUP93 | NUP98 | OBSCN |
| OGDH | OMD | OPCML | OR11G2 | OR2T4 | OR4A15 | OR4C6 | OR5L2 | OR6F1 | P2RY8 |
| P4HB | PABPC1 | PABPC3 | PAG1 | PAK1 | PAK3 | PASK | PAX3 | PAX7 | PC |
| PCDH18 | PCSK6 | PCSK7 | PDCD11 | PDE4DIP | PDGFB | PDILT | PER1 | PGR | PHF1 |
| PHF6 | PIK3C2A | PIK3C2B | PIK3C2G | PIK3C3 | PIM1 | PKD1L2 | PKHD1 | PLAG1 | PLCB1 |
| PLCG1 | PLCG2 | PLK1 | PLXNA1 | PLXNB2 | PNRC1 | POLQ | POM121 | POM121L12 | POU2AF1 |
| PPM1D | PPP1R17 | PPP6C | PRDM16 | PREX2 | PRF1 | PRKAA1 | PRKCB | PRKCI | PRKDC |
| PRRX1 | PRX | PSG2 | PSIP1 | PSMB1 | PSMB5 | PTGS1 | PTGS2 | PTPN13 | PTPN2 |
| PTPRB | PTPRK | PTPRO | PTPRS | PTPRT | PTPRU | RAB35 | RAC2 | RAD21 | RAD54B |
| RANBP2 | RASA1 | RASGRP1 | RBL1 | REL | RELN | RFC1 | RGS3 | RHEB | RHOH |
| RHOT1 | RIT1 | RNASEL | ROBO1 | ROBO2 | ROBO3 | ROCK1 | RPGR | RPS6KB1 | RPS6KB2 |
| RSPO2 | RSPO3 | RUNX1T1 | RUNX2 | RXRA | RYR1 | RYR2 | SBDS | SCUBE2 | SDC4 |
| SEC31A | SEMA3A | SEMA3E | SEMA6A | SERPINA7 | SETBP1 | SETDB1 | SF1 | SF3A1 | SFPQ |
| SGCZ | SGK1 | SH2B3 | SH2D1A | SH3PXD2A | SHH | SI | SIN3A | SLC16A1 | SLC1A2 |
| SLC22A16 | SLC22A18 | SLC22A2 | SLC22A3 | SLC34A2 | SLCO1B3 | SLIT1 | SLIT2 | SMARCD1 | SMARCE1 |
| SMC1A | SMC1B | SNCAIP | SNTG1 | SNX29 | SOD2 | SOS1 | SOX10 | SOX17 | SPEN |
| SPRR3 | SPSB4 | SPTA1 | SRD5A2 | SRGAP1 | SRGAP3 | SRSF2 | SRSF7 | STAG1 | STAT1 |
| SUCLG1 | SUCLG2 | SULT1A1 | SUZ12 | SVEP1 | SYNCRIP | SYNE1 | TAF1 | TAF15 | TAF1L |
| TAL1 | TBL1XR1 | TBX15 | TBX22 | TCEB1 | TCF12 | TCF3 | TCF4 | TCL1A | TEC |
| TENM3 | TERT | TET1 | TFDP1 | TFDP2 | TFE3 | TGFBR1 | THBS2 | TJP1 | TLE1 |
| TLL2 | TLR4 | TLX3 | TMEM132D | TNFSF11 | TNN | TP53BP1 | TP63 | TP73 | TPM3 |
| TPR | TRAF2 | TRAF7 | TRIM24 | TRIM58 | TRIO | TRPC5 | TRRAP | TSHZ2 | TSHZ3 |
| TTF1 | TUBA3C | TUBB3 | TUSC3 | TXNIP | TYMS | TYR | UBE2D2 | UBR5 | UGT1A1 |
| UMPS | UPF3B | USH2A | USP6 | USP8 | VEZF1 | VIM | VTCN1 | WASF3 | WDR90 |
| WDTC1 | WHSC1 | WHSC1L1 | WIPF1 | WNK1 | WNT5A | WSCD2 | WWOX | WWP1 | WWP2 |
| XIAP | XPC | XRCC1 | XRCC3 | YAP1 | YY1AP1 | ZBTB16 | ZC3H11A | ZFHX3 | ZFP36L1 |
| ZFP36L2 | ZFPM2 | ZIC3 | ZNF217 | ZNF384 | ZNF521 | ZNF638 | ZNF750 | ZNF804B | |
| **36 HRR genes analyzed** | | | | | | | | | |
| ATM | ATR | ATRX | BAP1 | BARD1 | BLM | BRCA1 | BRCA2 | BRIP1 | CDK12 |
| CHEK1 | CHEK2 | C11orf30 | ERCC1 | FAM175A | FANCA | FANCC | FANCD2 | FANCE | FANCF |
| FANCG | FANCL | FANCM | MRE11 | NBN | PALB2 | RAD50 | RAD51 | RAD51B | RAD51C |
| RAD51D | RAD52 | RAD54L | RECQL | RECQL4 | WRN | | | | |
